# Can non-human primates serve as models for investigating dengue disease pathogenesis?

**DOI:** 10.3389/fmicb.2013.00305

**Published:** 2013-10-11

**Authors:** Kristina B. Clark, Nattawat Onlamoon, Hui-Mien Hsiao, Guey C. Perng, Francois Villinger

**Affiliations:** ^1^Department of Pathology and Laboratory Medicine, Emory Vaccine Center, Emory University School of MedicineAtlanta, GA, USA; ^2^Office of Research and Development, Faculty of Medicine Siriraj Hospital, Mahidol UniversityBangkok, Thailand; ^3^Center of Infectious Disease and Signaling Research, National Cheng Kung UniversityTainan, Taiwan; ^4^Department of Microbiology and Immunology, College of Medicine, National Cheng Kung UniversityTainan, Taiwan

**Keywords:** dengue virus, disease pathogenesis, non-human primate, hemorrhage, platelet, bone marrow, platelet-lymphocyte aggregate, rhesus macaque

## Abstract

Dengue Virus (DV) infects between 50 and 100 million people globally, with public health costs totaling in the billions. It is the causative agent of dengue fever (DF) and dengue hemorrhagic fever/dengue shock syndrome (DHF/DSS), vector-borne diseases that initially predominated in the tropics. Due to the expansion of its mosquito vector, *Aedes* spp., DV is increasingly becoming a global problem. Infected individuals may present with a wide spectrum of symptoms, spanning from a mild febrile to a life-threatening illness, which may include thrombocytopenia, leucopenia, hepatomegaly, hemorrhaging, plasma leakage and shock. Deciphering the underlining mechanisms responsible for these symptoms has been hindered by the limited availability of animal models that can induce classic human pathology. Currently, several permissive non-human primate (NHP) species and mouse breeds susceptible to adapted DV strains are available. Though virus replication occurs in these animals, none of them recapitulate the cardinal features of human symptomatology, with disease only occasionally observed in NHPs. Recently our group established a DV serotype 2 intravenous infection model with the Indian rhesus macaque, which reliably produced cutaneous hemorrhages after primary virus exposure. Further manipulation of experimental parameters (virus strain, immune cell expansion, depletion, etc.) can refine this model and expand its relevance to human DF. Future goals include applying this model to elucidate the role of pre-existing immunity upon secondary infection and immunopathogenesis. Of note, virus titers in primates *in vivo* and *in vitro*, even with our model, have been consistently 1000-fold lower than those found in humans. We submit that an improved model, capable of demonstrating severe pathogenesis may only be achieved with higher virus loads. Nonetheless, our DV coagulopathy disease model is valuable for the study of select pathomechanisms and testing DV drug and vaccine candidates.

## Introduction

Dengue Virus (DV), the causative agent of dengue fever (DF), is the most important vector-borne human pathogen, infecting between 50 and 100 million people annually (Who, 2012). Moreover, DF is an escalating human problem that is increasingly spreading across the globe and extending in seasonality. This recent growth is attributed to the expansion in the niche of the virus-transmitting vectors, primarily *Aedes albopictus* and *Aedes aegypti* (Who, 2011). Thanks to the lack of vector control, increased human travel and global warming, DF, once considered a tropical disease, may reach a worldwide distribution.

The majority of DV infections are asymptomatic or mild, but for about a quarter of infected people, disease may present as an illness that is indistinguishable from other febrile diseases or as DF with minor hemorrhagic abnormalities, bone pain, decreases in platelet counts and leucopenia, the most common form of disease. Rarely, people present with the severe forms—dengue hemorrhagic fever (DHF) in which patients display hematomas with a marked thrombocytopenia or extremely low platelet counts and dengue shock syndrome (DSS), a disease similar to DHF but including plasma leakage/heme concentration, pleural effusion and the increased risk of multi-organ failure (Who and Tdr, 2009). Other symptoms (abnormal bleeding, melena, hepatomegaly, vomiting, etc.) have also been reported (Cobra et al., 1995). The majority of severe DHF/DSS cases in endemic countries occur in healthy adolescents 10–24 years of age (Tsai et al., 2012). Early identification of the causative agent and immediate hydration therapy with extensive monitoring of symptoms is important for resolving symptoms and preventing fatal outcomes (Who and Tdr, 2009). There is currently no targeted therapy to modulate disease severity of those most vulnerable.

It has been surmised that factors such as genetic susceptibility, developmental stage, environmental exposures and immune system programming induced by previous infections may predispose young adults to more severe disease (Halstead et al., 2007). Epidemiological data obtained from endemic countries reveal that DHF/DSS most often occurs in people with a secondary antibody response, which has led many to champion the antibody-dependent enhancement (ADE) of infection hypothesis (Endy et al., 2004; Fox et al., 2011). ADE proponents believe that weakly specific, cross-reacting antibodies facilitate virus entry into permissive cells, increasing titers and thus, disease. Though some ADE proponents suggest that dengue-specific antibody increases immunopathology without necessarily enhancing virus replication (Markoff et al., 1991; Lei et al., 2001; Oishi et al., 2003). On the contrary, many reports have failed to demonstrate an association of DHF/DSS with secondary infection (Murgue et al., 1999, 2000; Cordeiro et al., 2007; Guilarde et al., 2008; Libraty et al., 2009; Meltzer et al., 2012). A better association may exist between virus titers and disease severity (Murgue et al., 2000; Libraty et al., 2002). Despite the uncertainty over ADE, it is required that this potential risk factor be considered during the formulation of all vaccines under development (Who, 2011). Standard preventative modalities incorporate representative antigens of each serotype in effort to simultaneously induce protection to all four DV strains.

In the past, vaccines were designed without an exact understanding of the mechanism(s) responsible for disease pathogenesis; this was done by selecting for candidates that reduced viremia and elicited strong antibody responses (Cox, 1953; Togo, 1964). Unfortunately this approach has failed with DV, a pathogen that does not elicit strong humoral immunity in natural infections. Neutralizing antibody to DV can be elicited in a variety of primates (chimpanzees, cynomolgus macaques, African green monkeys, etc.) after primary infection, but they are often weak and short-lived (Scherer et al., 1978; Bernardo et al., 2008; Martin et al., 2009). In addition, protection from viremia has been reported in rhesus macaques that develop poor neutralizing antibody titers (Scott et al., 1980; Putnak et al., 1996) and after the response waned (Raviprakash et al., 2000). Interestingly, some evidence suggests that humans may also be protected from disease during high viremia without ever developing specific antibodies (Stramer et al., 2012; Perng and Chokephaibulkit, 2013); these observations raise concern that neutralizing antibody quantification is not the best approach to evaluate vaccine efficacy.

A more thorough understanding of the mechanisms contributing to disease and protection in humans is clearly needed to accelerate progress toward better drug and vaccine candidates. Severe disease is known to arise after the clearance of viremia, suggesting that DHF/DSS and lethality are more likely immune than viral-mediated (Who and Tdr, 2009). In fact, immune activities elicited via antibodies (Saito et al., 2004), complement (Avirutnan et al., 2006) and T cells (Green et al., 1999) have been associated with disease in human studies. Importantly, the delay in severe disease presentation until late after infection limits our ability to interrogate early events that set the stage for immunopathogenesis. Thrombocytopenia, plasma leakage, and coagulation abnormalities appear to be the critical phenomena to prevent in patients, but the events preceding these phenomena have been incompletely elucidated. Carefully controlled experiments performed in relevant animal models are needed to explore the dynamics of hematological dysfunction and other factors potentially involved in dengue disease. Unfortunately an adequate animal model that is capable of recapitulating human disease is largely unavailable.

## Development of DV infection animal model systems

The search for animal model systems began in the early 1900s, far before the availability of cell culture techniques to propagate or quantify virus stocks. Pathogens had to be amplified in animals that were permissive and quantified by mortality studies. Unfortunately none of the animals tested (hamster, mouse, rat, lizard, etc.) ever displayed signs of disease, limiting the progress in studying DV (Simmons et al., 1931). The research that was conducted often involved virus propagation in human volunteers, who suffered from typical DF (Simmons et al., 1931). Eventually, a young suckling mouse model inoculated intracranially with DV that displayed mild disease was developed (Sabin and Schlesinger, 1945). This model was quite limited, with paralysis observed only after 3–4 weeks in 10–20% of the mice, but this provided a starting point for virus adaptation and lead to the first small animal infection model.

## Mouse model

There are a number of mouse breeds that have been employed in DV investigations–wildtype, engrafted-SCID, AG129, RAG-hu, and the NOD/SCID/IL-2Rγ/human CD34 transplant or humanized mouse (Lin et al., 1998; Kuruvilla et al., 2007; Zhang et al., 2007; Mota and Rico-Hesse, 2011; Zompi et al., 2011). AG129 mice have been the most commonly utilized strain; they are highly susceptible to dengue, replicate virus to high titers and display vascular leakage (Shresta et al., 2006; Zompi and Harris, 2012). The NOD/SCID/IL-2Rγ mice reconstituted with human CD34+ cells are infrequently used but have the greatest potential as future mouse models. These animals demonstrate several symptoms of human disease (fever, erythema, thrombocytopenia) (Mota and Rico-Hesse, 2011; Cox et al., 2012).

However, the symptomatology observed with inbred, immune-compromised mice differs from that seen in humans, likely because of the susceptibility of various cell lineages and the extensive differences in immune system dynamics (Nussenblatt et al., 2010). AG129 mice predominantly display neurological symptoms and splenomegaly (Schul et al., 2007; Zompi and Harris, 2012) and engrafted-SCID mice present with paralysis (Zompi and Harris, 2012). While the humanized mouse may be the closest to replicating patient pathology, there still remain a few caveats to using this model. Challenges involved in humanized mouse preparation and data interpretation are compounded by the considerable mouse-to-mouse variation observed (Akkina et al., 2011). Additionally this mouse model, with murine stroma and endothelium, cannot completely mimic the immune response of humans. A number of mechanisms suspected to play critical roles in dengue pathology are differentially regulated in these mice. Processes that are dependent on stromal cell interactions, such as B lymphocyte maturation and specific antibody production (Akkina, 2013), and involve endothelial microparticle signaling, such as the coagulation cascade (Mairuhu et al., 2003; Lynch, 2007), may unfold differently in these mice and lead to alternative outcomes. The human CD34+ engrafted mouse model system can provide a great starting point in interpreting important biological processes involved in human DV disease but results will still need to be confirmed in non-human primate (NHP) species.

## Non-human primate (NHP) models

It has been hypothesized that the close genetic relationship between primates and humans and the presence of a comparable immune responses make NHPs the best models for studying DV. While this may be, NHPs have been particularly unreliable at modeling DV pathology, producing mild symptoms at best (Scherer et al., 1972; Halstead et al., 1973b). Monkeys thus far appear to be incapable of succumbing to life-threatening DV disease. However, several Old and New World primate species are in fact permissive to experimental DV infection (Scherer et al., 1978; Schiavetta et al., 2003; Onlamoon et al., 2010; Yoshida et al., 2012). A recently published review detailed the characteristics of viremia in many of these species (Hanley et al., 2013). Table 1 summarizes the pathology and immunopathology observed thus far in ~20 NHP species from 15 different genera.

**Table 1:** **Summary of *in vivo* DV studies**.

**Primate**	**Route**	**Strain**	**Type**	**Virus stock^a^**	**Dose^b^**	**Infected**	**Viremia^c^**	**Findings (source)**
*Macaca mulatta*	iv, sc	ND	ND	Humans	ND	ND	ND	No disease, leucopenia (Lavinder and Francis, 1914)
*Macaca cyclopis*	sc, iv, ip	ND	ND	Humans	ND	ND	ND	No disease (Koizumi and Tonomura, 1917)
*Macaca mulatta*	NI	ND	ND	Humans	ND	Yes	ND	Animal chilly and morose, rash on chin, and throat (Chandler and Rice, 1923)
*Macaca fascicularis*	sc	ND	ND	Humans	ND	Yes	ND	First to demonstrate unquestionably that some primates were permissive to DV
*Cercopithecus callitrichus*		ND	ND	Humans	ND	Yes	ND	infection but that they are asymptomatic
*Papio* spp.		ND	ND	Humans	ND	No	ND	
*Cercocebus* spp.		ND	ND	Humans	ND	No	ND	(Blanc et al., 1929)
*Macaca mulatta*	sc, mi	ND	ND	Humans, mosquitoes	ND	No	ND	
*Macaca fascicularis philippinensis*	sc, mi, ic	ND	ND	Humans, mosquitoes	ND	Yes	ND	No fever, some leukopenia and lymphocytosis, demonstrated transmission of DV from primates to humans through mosquitoes
Macaca fascicularis fusca^*^	sc, mi	ND	ND	Humans, mosquitoes	ND	Yes	ND	(Simmons et al., 1931)
Pan troglodytes^*^	sc, id	Hawaiian	NI	Human	ND	Yes	ND	Mild fever (101°F) (Paul et al., 1948)
*Homo sapiens*	id	NI	NI	Human	1^d^	Yes	+	Low dose gave multiple patterns of disease: (1) unmodified attack, (2) short febrile illness without rash or 3) no illness but partial immunity
	id				10^d^	Yes	+	Progression of symptoms:(1) edema and erythema, (2) fever, (3) maculopapular eruptions with sparing at the site of the original skin lesion
	into scars				Conc. human serum	Yes	+	Unmodified dengue
	eye				2E5^d^	Yes	+	Typical dengue
	eye				1E4^d^	No	−	No disease or immunity
	in				1E6^d^	Yes	+	Unmodified dengue or mild rash
	in				1E4^d^	No	−	No disease or immunity (Sabin, 1952)
*Cebus capucinus*	sc or ip	Hawaiian, NGC	DV1, DV2	Human	ND	Yes	+	No overt signs of illness
*Ateles geoffroyi*						Yes	+	
*Ateles fusciceps*						Yes	+	
*Alouatta palliata*						Yes	+	
Callithrix geoffroyi^*^						Yes	ND	
*Saimiri oerstedii*						Yes	ND	
*Aotus trivirgatus*						Yes	ND	(Rosen, 1958)
*Hylobates lar*	sc	BKM725-67	DV1	LLC-MK2	800	Yes	+	Fever and hemorrhagic manifestations occurred but were associated with acute
		BKM1179-67	DV1		800			Lymphomatous leukemia, no correlation between antibody titers to
		BKM1749	DV2		1.6E3			DV and protection from viremia
		24969	DV3		6.6E2			
		KS168-68	DV4		5E3			(Whitehead et al., 1970)
*Saimiri sciureus*	sc	Hawaii	DV1	Mice	1E6.4^e^	Yes	+	Some fever in DV1 infection,
		16007	DV1	LLC-MK2	1E5.7	Yes	−	No platelet, hematocrit or leukocyte count changes
		NGC	DV2	Mice	1E6.7^e^	Yes	+	
		NGC	DV2	mosquitoes	1E2.5	Yes	+	
		16681	DV2	LLC-MK2	1E5.5	Yes	−	
		Pak-20	DV3	LLC-MK2	1E3.4	Yes	50	
		16562	DV3	LLC-MK2	1E5.7	Yes	−	
		4328S	DV4	LLC-MK2	1E3.9	No	−	
*Saguinus oedipus*	sc	Hawaii	DV1	Mice	1E6.4^e^	Yes	+	Brief fever in DV1 infection
		NGC	DV2	Mice	1E6.7^e^	Yes	+	
		NGC	DV2	Mosquitoes	1E2.5	Yes	+	
		H87	DV3	Mice	1E5.8	Yes	−	
		Pak-20	DV3	LLC-MK2	1E3.4	Yes	−	
		H241	DV4	Mice	1E6.6^e^	No	−	
*Saimiri sciureus*	in	Hawaii	DV1	Mice	1E6.4^e^	Yes	ND	No disease reported
		NGC	DV2	Mice	1E5^e^	No	ND	
		NGC	DV2	mosquitoes	1E2.5	No	ND	
		Pak-20	DV3	LLC-MK2	1E2.1	No	ND	
		H-241	DV4	Mice	1E6.6^e^	No	ND	
*Saguinus oedipus*	in	NGC	DV1	Mice	1E5.3^e^	Yes	+	
		H87	DV3	Mice	1E6.2^e^	Yes	ND	
		Pak-20	DV3	LLC-MK2	1E2.7	No	ND	
		H241	DV4	Mice	1E5.6^e^	No	ND	
*Aotus trivirgatus*	in	Hawaii	DV1	Mice	1E5.7^e^	No	ND	
		NGC	DV2	Mice	1E6.6^e^	Yes	ND	
		Pak-20	DV3	LLC-MK2	1E2.1	Yes	ND	(Scherer et al., 1972)
*Macaca mulatta* (Indian)	sc	16007	DV1	LLC-MK2	5E5	Yes	1.7E3	Lymphadenopathy in DV1, 2, & 4, rare hemorrhaging in DV1& 4, leucopenia
		16681	DV2	LLC-MK2	5E5	Yes	4.8E2	In DV2 & 4, lymphocytosis was common
		16562	DV3	LLC-MK2	5E5	Yes	+	Thrombocytopenia in 21–33% of animals with all serotypes
		4328S	DV4	LLC-MK2	5E5	Yes	2.8E2	Complement decreases in secondary DV2, no change in behavior, eating or prothrombin
*Macaca*	sc, id	16007	DV1	LLC-MK2	NI	Yes	−	No disease
*fascicularis*		16681	DV2		NI	Yes	−	
fascicularis^*^		16562	DV3		NI	Yes	−	
		4328S	DV4		NI	Yes	−	
*Chlorocebus*	sc, id	16007	DV1	LLC-MK2	1E5	Yes	+	No disease
aethiops^*^		16681	DV2		1E5	Yes	+	
		16562	DV3		1E4.5	Yes	+	
*Erythrocebus*	sc, id	16007	DV1	LLC-MK2	NI	Yes	+	No disease
*patas*		16681	DV2		1E5	Yes	+	
		16562	DV3		1E4.5	Yes	−	
		4328S	DV4		1E3.3	Yes	−	(Halstead et al., 1973a,b)
*Macaca mulatta*	sc	16007	DV1	LLC-MK2	1.2E5	Yes	350	Lymphadenopathy, virus distribution after sc injection indicated that most virus did not move far from the inoculation site, day after viremia virus was distributed widely throughout skin (Marchette et al., 1973)
		16681	DV2		2E6	Yes	443	
		16562	DV3		1E5	Yes	40	
		4328S	DV4		1E6	Yes	1085	
*Pan troglodytes*	id, sc	49313	DV1	Mosquitoes	1E3.1	Yes	1E6.6^g^	Nasal discharges and lymphadenopathy
		NC38	DV2	Humans	1E3.6	Yes	1E5.6^g^	Symptoms found in individual animals
		49080	DV3	Mosquitoes	1E2.7	Yes	1E5.2^g^	Splenomegaly, leucopenia
		17111	DV4	Mosquitoes	1E2.8	Yes	1E6^g^	Hemorrhage, shaking chill, lethargy (Scherer et al., 1978)
*Macaca mulatta*	sc	16681	DV2	LLC-MK2	1E5	Yes	1E5.7	Cyclophosphamide treatment caused chronic infection, 3/9 died, internal hemorrhaging, enlarged kidney, severe acute proliferative glomerulonephritis, pleural effusion, passively transferred antibody aided viral clearance (Marchette et al., 1980)
*Macaca mulatta*	sc	PR-159	DV2	FRhL	5.6	Yes	ND	No disease
		H-241	DV4		1.44			(Kraiselburd et al., 1985)
*Macaca mulatta & Macaca fascicularis*	is, im, it	16007	DV1	PDK	2.5E5	Yes	ND	Mild neurovirulence (Angsubhakorn et al., 1987)
*Aotus nancymae*	sc	Western Pacific 74	DV1	NI	2E4	Yes	+	Pathology more pronounced in DV1, mild leucopenia, changes in attitude and appetite
		S16803	DV2					Changes in fecal consistency, 2/20 became lethargic
		CH53489	DV3					Common symptoms: lymphadenopathy, nasal discharges and splenomegaly (Schiavetta et al., 2003)
		341750	DV4					
*Aotus*	sc	IQT6152	DV1	NI	1E4	Yes	+	No disease
*nancymae*		IQT2124	DV2				−	
		OBS8041	DV2				+	(Kochel et al., 2005)
*Macaca*	sc	60305	DV1	Vero	1E5	Yes	1E1.6	No disease
*mulatta*		16007	DV1	Vero	1E5	Yes	1E2.4	
		16007	DV1	C6/36	1E5	Yes	1E1.9	
		40247	DV2	C6/36	1E5	Yes	1E3.6	
		44/2	DV2	Vero	1E5	Yes	1E2.9	
		H87	DV3	Vero	1E5	Yes	1E2.7	
		16562	DV3	Vero	1E5	No	−	
		74886	DV3	C6/36	1E5^f^	Yes	1E2.2	(Freire et al., 2007)
*Macaca fascicularis*	sc	40514	DV1	NI	1E6.4^f^	Yes	400^f^	No disease, characterized T-cell and neut antibody cross-reactivity, no changes in
		28128	DV4		1E6.2^f^		20^f^	IFN-γ, TNFα, IL4, IL8, IL10 transcription during infection (Koraka et al., 2007)
*Macaca mulatta*	sc	Western Pacific 74	DV1	NI	1E4	Yes	ND	No disease, increases in AST, transcriptional upregulation of
								ISGs, OASs, Mxs, etc., no increases in cytokine gene expression (Sariol et al., 2007)
*Chlorocebus aethiops sabaeus*	sc	SB8553	DV2	NI	1E6	Yes	+	No fever or lymphomegaly, no changes in behavior or weight, no respiratory, digestive or nervous system disturbances, lower inoculum titers gave prolonged viremia and better neut antibody responses (Martin et al., 2009)
*Macaca mulatta* (Indian)	iv	16681	DV2	Vero	1E7	Yes	~8E3	Consistent hemorrhaging in 9/9 animals, decline in platelet count and leucopenia, elevated thrombin-antithrombin, D-dimers, ALT, and CK, no increases in hematocrit, prothrombin or activated PTT (Onlamoon et al., 2010)
*Callithrix jacchus*	sc	02–17/1	DV1	C6/36	3.5E7	Yes	5E5^h^	No disease
		DHF0663	DV2		6.7E7		1.6E7^h^	Found differing NK, NKT, and niave effector memory and central T-cell kinetics during DV infection with different strains
		DSS1403	DV3		4.5E6		5.5E4^h^	
		05-40/1	DV4		1.5E6		2.5E4^h^	
		Jam/77/07	DV2		1.2E5		2.8E6^h^	
		Mal/77/08	DV2		1.9E5		9.6E6^h^	(Omatsu et al., 2011; Yoshida et al., 2013)
*Homo sapiens*	sc	45AZ5	DV1	FRhL	2E3	Yes	+	CD8+T-cell-dervied IFN-γ associated with protection from fever and viremia, sIL-R2α correlated with disease onset and severity, PBMC-derived TNF-α, IL-2, 4, 5, 10 did not correlate with protection or disease (Gunther et al., 2011; Sun et al., 2013)
		CH53489	DV3	FRhL	1E5			
*Macaca nemestrina*	sc	98900645	DV3	C6/36	1E7-1E8	Yes	62.94	Inoculation route influenced virus-tissue distribution
	id						47.98	Minimal hepatitis
	iv						58.62	(Pamungkas et al., 2011)
*Saguinus midas and Saguinus labiatus*	sc	DHF0663	DV2	C6/36	6.7E7	Yes	2.7E6^h^	No disease, CD16+ NK cell depletion did not alter virus replication or pathogenesis
	iv						2E7^h^	(Yoshida et al., 2012)
*Macaca mulatta* (Indian)	sc	NGC	DV2	NI	1E5	Yes	257	Day 14 PI showed the highest levels in T-cell activation, Anti-NS1, 3, & 5 T-cell responses were characterized (Mladinich et al., 2012)
*Macaca mulatta* (Chinese)	iv, sc	16681	DV2	Vero	1E7	Yes	+	Hemorrhaging in 50% of iv inoculated primates (unpublished)

a Cell type or organism in which DV stock was propagated;

b Highest inoculum dose is given when there were variable doses;

c Titers given when available;

d HID;

e MLD50orMLD50/ml;

f TCID50orTCID50/ml;

g MID50/ml;

h RNA/ml; +/−, indicates presence or absence of viremia, ic, intracardial; mi, mosquito inoculation; iv, intravenous; sc, subcutaneous; id, intradermal, ip, intraperitoneal; in, intranasal; im, intramuscular; is, intraspinal; it, intrathalmic; NI, not indicated; ND, not determined; MID50, mosquito infectious dose 50; TCID50, tissue culture infectious dose 50; MLD50, suckling mouse intracranial lethal dose 50; HID, human minimal infectious dose;

* indicatesspeciesnamechange.

The most consistent pathological finding in these animals have been lymphadenopathy of the inguinal and auxiliary lymph nodes (Halstead et al., 1973a; Marchette et al., 1973; Scherer et al., 1978; Schiavetta et al., 2003). In one species, *Chlorocebus aethiops sabaeus*, the absence of lymphomegaly (Martin et al., 2009) and in a few reports, splenomegaly (a rare symptom in humans) were noted (Scherer et al., 1978; Schiavetta et al., 2003). Fever is a valid parameter to assess, but its recording in DV-infected primates is logistically difficult, and is therefore rarely reported (Scherer et al., 1972). NHPs in general have higher body temperatures and greater variability than human bodies (Scherer et al., 1972; Fuller et al., 1985), so unless readings are measured on awake animals by telemetry, the anesthesia used profoundly alters the body's temperature, making accurate readings impossible (Baker et al., 1976). Another human dengue symptom, cutaneous rashes, are not commonly observed in primates but may be underreported; also tourniquet tests are never performed on primates to assess capillary fragility. Behavioral changes, like lethargy, have been documented in only a few studies (Chandler and Rice, 1923; Scherer et al., 1978; Schiavetta et al., 2003). In general, primates kept and bred in captivity rarely display overt disease.

Despite the low incidence of pathology observed in these studies, dengue infections in primates share many characteristics with human disease. The onset and duration of viremia is similar to humans, or about 3–6 days starting from the second day after inoculation (Freire et al., 2007; Koraka et al., 2007). Leucopenia has been observed (Onlamoon et al., 2010). Thrombocytopenia has never been captured in NHPs, likely because of their naturally high platelet counts, but moderate platelet decreases have been document in *M. mulatta* (Halstead et al., 1973a; Onlamoon et al., 2010). A DV-induced reduction of dengue-specific antibodies during the early phases of secondary homologous infection, a phenomenon observed in viremic patients, has been seen in marmosets (Omatsu et al., 2011). The anti-dengue antibodies that are elicited in primates are highly cross-reactive against other closely related flaviviruses (Scherer et al., 1978). DV infection of monkeys elicits a vigorous innate response (Sariol et al., 2007) leading to activation and marked shifts in circulating subsets of T, NK, and NK-T cells in the marmoset model (Yoshida et al., 2013). The role of DV specific cell-mediated responses in NHP models has received relatively less attention, although some studies reported recognition of non-structural proteins in addition to viral components by both CD4+ and CD8+ T cells (Koraka et al., 2007; Mladinich et al., 2012). However, such responses have been difficult to detect in immunized monkeys, even in those that show protection from challenge (Chen et al., 2007; Porter et al., 2012).

The similarities observed in these studies imply that primates may present with more suitable symptoms than mouse models upon further manipulation. A comparison of the benefits to using the NHP and murine animal models is given (Table 2). Several strategies to improve the NHP model may be explored—for instance increasing the number of permissive cells or altering the immune environment. Here we discuss boosting viremia with different virus delivery strategies.

**Table 2:** **Relative advantages in using primate and murine model systems to study DV disease**.

	**Primate models**	**Murine models**
Ease of use/cost	−	+
Susceptibility to human DV strains	+	−
Mimic human viremia	(+) reduced	+
Mimic human immune responses	+	−
**MODEL HUMAN DISEASE**		
Fever	−	CD34-engrafted humanized mouse
Hemorrhages	Indian rhesus monkey	CD34-engrafted humanized mouse, C57BL/6
Platelet count reduction	Indian rhesus monkey	CD34-engrafted humanized mouse
Hepatomegaly	−	Balb/c
Pleural effusion	−	−
CNS disease^*^	−	+
DHF/DSS	−	−
Lethality	−	+

+, commonly present; −, absent;

* Rarely observed in human dengue infections.

## Virus delivery

Only a limited number of studies have attempted determining the infectious dose delivered during natural dengue infection. One study suggests the amount of DV transmitted by *A. aegypti* ranges from 1 × 10^4^ to 1 × 10^5^ (Gubler and Rosen, 1976). However, there are disagreements over the best methods to conduct such studies; the controversial points include mosquito species, generation number, feeding strategy, infection method, incubation temperature and length, virus strain and technique used to quantify transmitted virus. All these variables have the potential to affect the infection dynamics and alter the conclusions of the study (Chamberlain et al., 1954; Grimstad et al., 1980; Mellink, 1982; Watts et al., 1987; Colton et al., 2005; Smith et al., 2005). Some studies have suggested levels as high as 1 × 10^8.7^ genome equivalents or almost 1 × 10^7^ PFUs can be transmitted, though rarely (Colton et al., 2005; Styer et al., 2007). Currently we know as few as 1000 PFUs can cause viremia and disease symptoms in humans (Sun et al., 2013). Ultimately the natural inoculum dose is more suggestive of the amount of virus needed for continual DV transmission *in vivo* and does not necessarily reflect the quantity required for disease induction. Viremia levels and disease may be less dependent on inoculum size and more contingent on host-pathogen interactions. These matters should be considered when modeling DV infection in animals.

Virus delivery to the proper tissues is important for inducing the appropriate interactions with the host and promoting disease presentation. DV deposition is believed to occur exclusively by direct inoculation into the subcutaneous layer by mosquitoes. However, the subcutaneous infection route does not promote adequate virus dissemination (Marchette et al., 1973; Pamungkas et al., 2011). Potentially the virus is restricted by less frequent encounters with migrating cells and immobilization by attachment to extracellular matrix proteins (Anez et al., 2009). Consider that mosquito feeding involves the probing of all layers of skin, including the cutaneous layer and capillaries, to find a blood meal. These tissues are an integral part of the arbovirus-vector lifecycle and are frequently evaluated in transmission studies (Chamberlain et al., 1954; Styer et al., 2007). Virus injected directly into these tissues have better access to and faster dissemination throughout the body, affording the virus more opportunities to rapidly reach distant target cells (Pamungkas et al., 2011). Additionally, pathology induction is likely promoted by rapid viral dissemination and replication in distant cells and organs. This assumption led us to hypothesize that an intravenous infection strategy would favor wide dissemination and allow for rapid simultaneous replication of virus in various tissues, invoking a more pronounced innate immune response, potentially reflective of the human immune environment during high viremia. Although the kinetics of viremia did not markedly differ between subcutaneous and intravenous DV2 infection (Onlamoon et al., 2010; Omatsu et al., 2011), it will be critical to delineate the overall kinetics of DV dissemination to and replication in various tissues and how this relates to the induction of symptoms.

## Rhesus macaque model of coagulopathy

Only a few NHP dengue investigations have reported rashes post-infection (PI) (Lavinder and Francis, 1914; Halstead et al., 1973b; Onlamoon et al., 2010). In most of these studies, hemorrhaging was a rare event. However, our group reported a reproducible coagulopathy disease model in the Indian rhesus macaque when 9 out of 9 monkeys inoculated intravenously with 1 × 10^7^ PFUs of DV2 (16681) displayed evidence of subcutaneous hemorrhage (Onlamoon et al., 2010). The viremia noted in these animals remained at the high end of the range typically reported in other NHP studies and were reached relatively consistently at early time points PI.

**Figure 1:**
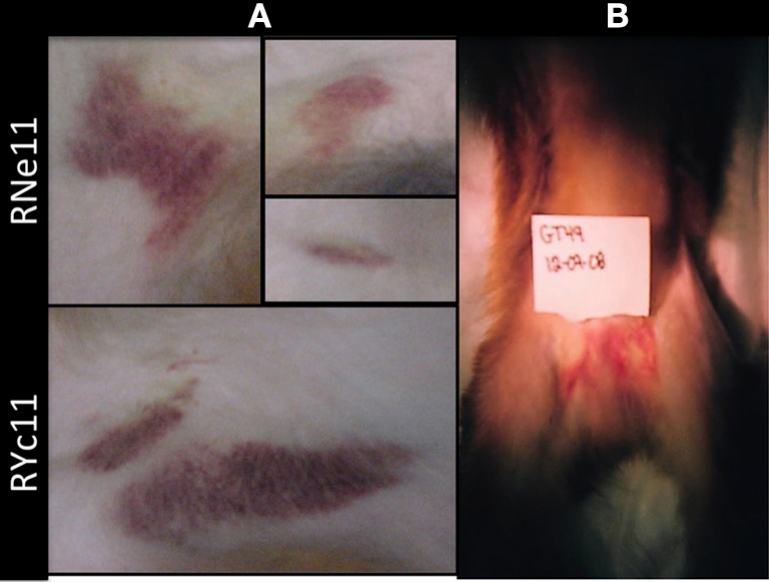
**Hematomas are seen in intravenously inoculated rhesus macaques. (A)** Indian rhesus macaques were injected intravenously with 1 × 10^7^ PFUs of DV2 16681 as previously reported (Onlamoon et al., 2010). Hematomas of various degrees of severity were present on Days 3 till 14 PI. Prominent ecchymoses were visible in two young male animals, RNell and RYc11, on Day 7. **(B)** Four Chinese rhesus macaques were injected intravenously (*n* = 2) or subcutaneously (*n* = 2) with 1 × 10^7^ PFUs of DV2 16681 strain. Hemorrhaging was only observed in 1 of 2 IV-injected monkeys (GT49), depicted in the picture above on Day 6 PI. No hematomas were observed in subcutaneously inoculated macaques.

The most prominent symptoms observed in our studies with the Indian rhesus macaque were cutaneous hemorrhages, starting at Days 3 and 4 PI and lasting as long as 10 days (Figure 1A) (Onlamoon et al., 2010). In a pilot study using Chinese rhesus macaques, disease presentation with the same virus was more modest, suggesting that these NHPs may be less susceptible to disease. Large hematomas developed in only one of the two primates infected intravenously with DV2 (Figure 1B).

The dynamics of various leukocyte subsets were followed longitudinally PI. Similar to human dengue, these animals experienced the typical leucopenia or a modest but consistent decrease in white blood cells that reached a nadir at Day 7 PI, but returned to normal levels by Day 10 (Onlamoon et al., 2010). Platelets also modestly decreased until Day 3, corresponding to the time of peak DV RNA load (Noisakran et al., 2012). While these leukocyte values did not fall out-of-range for macaques the changes were clearly noticeable and consistent. There was also a modest decrease in hematocrit, which resolved with the clearance of viremia at Day 7, in spite of continuous blood and bone marrow (BM) draws (Onlamoon et al., 2010).

A longitudinal monitoring of coagulatory parameters hinted that a number of features may be important for hemorrhage formation (Onlamoon et al., 2010). Increased time to clotting was noted during blood collection of some Indian rhesus macaques, indicating an increased susceptibility toward bleeding. However, thromboplastin and prothrombin times did not indicate abnormal clotting. Protein C and anti-thrombin III levels did not vary from pre-inoculation values, but they were predominantly in the high end of the reference range. Marked elevations were noted for D-dimers, TAT complexes and protein S, with peaks most consistently present on Days 5–10 PI, corresponding to the resolution of viremia. This data requires further confirmation with additional time points, more animals spanning various ages and other DV isolates. However, we submit that we might for the first time have a model to investigate coagulopathy similar to DHF, which can allow for better evaluation of preventative and therapeutic strategies to prevent pathogenesis, not just infection.

Interestingly, analysis of serum chemistry parameters indicated relatively modest changes for all parameters except creatine phosphokinase (CK), which was markedly elevated on Day 7 (Onlamoon et al., 2010). CK is a component in energy metabolism (with multiple isoenzymatic forms: MM, MB, and BB) that are altered in individuals with a number of different illnesses (Roberts and Sobel, 1973; Saks et al., 1978). Heightened levels of CK have been noted in Crimean Congo and Influenza patients (Middleton et al., 1970; Ergonul et al., 2004). Additionally, a recent report confirms elevation of this enzyme in dengue patients and suggests it is linked to muscle weakness/dysfunction during malaise (Misra et al., 2011). However, CK is a non-specific biomarker that is elevated in various conditions, and thus its diagnostic value is limited. Since these enzymes are quite highly elevated during DV infection, there could be a meaningful relationship between CK and disease. CK and creatine phosphates in combination are known as ADP scavengers and participate in modulating platelet activities, such as aggregation (Chignard et al., 1979; Chesney et al., 1982; Krishnamurthi et al., 1984; Jennings, 2009), which may consequently modulate immune cell activation/function and by extension, pathogenesis (Wong et al., 2013).

## Bone marrow (BM) targeting

**Figure 2:**
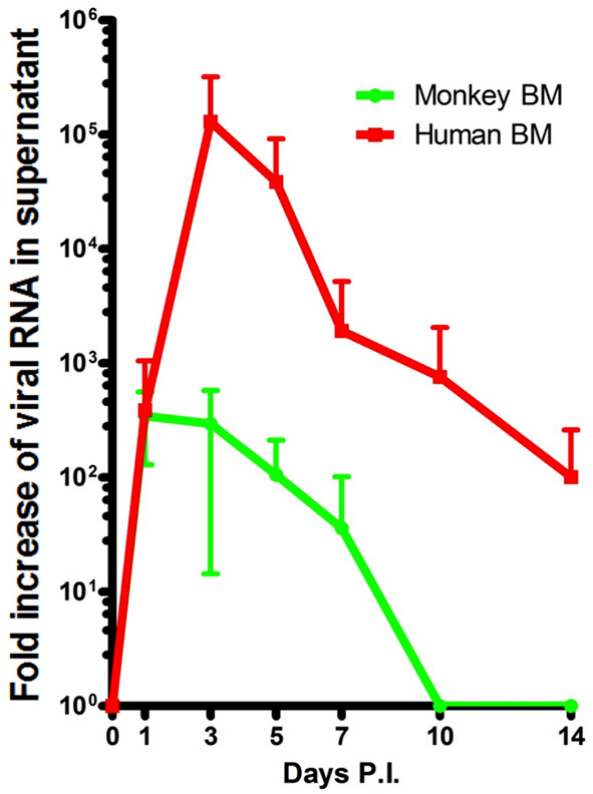
**Peak DV titers in rhesus macaque BMs is markedly lower than that of humans.** BMs were acquired and infected as previously described (Clark et al., 2012). Samples from Days 1 through 14 were quantified by realtime PCR. Human (red) and monkey (green) titers are depicted in RNA copy numbers per ml. The *in vitro* experimentation of whole BM indicates that human BM is able to produce far more virus than monkey BM. Titers appear to max out on average closer to Day 1 in monkey BM but reach their peak (~1000-fold higher) on Day 3 PI in humans.

The BM can be involved in hemodynamic defects; alterations in the BM environment may result in altered leukocyte function and contribute to pathogenesis (Wilson and Trumpp, 2006; Duffy et al., 2012). DV has long been known to alter hematopoiesis in human BM (Bierman and Nelson, 1965; La Russa and Innis, 1995). However, collecting BM aspirates from DV patients is contraindicated. Additionally, infections in patients can be misleading due to the variability in disease onset and the uncertainty of sample time points. Experimentation in animal models in which the induction of infection is known allows for better analysis in real time. Our rhesus monkeys were sampled for BM repeatedly on a rotating basis resulting in the collection of at least 3 samples at each time point spanning Days 1–14 PI. This has allowed for us to confirm that BM cellularity is indeed depressed during early acute DV infection (Noisakran et al., 2012). Aspirates were also monitored for the presence of DV in attempts to identify the initial cellular reservoirs of infection. While the general consensus is that DV targets phagocytes, such acquisition could be secondary to amplification in other cell types. *In vitro* both human and monkey BMs are permissive for DV replication, and similar to *in vivo*, peak titers differ by 1000-fold (Figure 2) (Clark et al., 2012). Characteristics of the early host cells were also evaluated in our model both *in vivo* and *in vitro* (Clark et al., 2012; Noisakran et al., 2012). Of interest DV antigen was primarily detected in CD41+ CD61+ cells during the first 3 days, followed by a gradual shift toward CD14+ phagocytes at later time points, coinciding with viral clearance (Clark et al., 2012). The results suggest that megakaryocytes represent the initial target of DV in BM, rather than a member of the monocytic lineage. Direct infection of these cells may account for the altered megakaryocyte composition (Nelson et al., 1964), impaired platelet function (Srichaikul and Nimmannitya, 2000; Cheng et al., 2009) and the incidence of platelet phagocytosis observed in previous studies (Nelson et al., 1966; Honda et al., 2009; Onlamoon et al., 2010). Platelet activation and function during the course of infection has been under-investigated but may be critical for unraveling the mechanisms responsible for dengue pathology.

## Platelet activities

The role of platelets in the crafting of the immune response is imperfectly defined and only recently becoming recognized (Klinger and Jelkmann, 2002; Ombrello et al., 2010). These anucleated cells are able to associate with and deliver signals to other lineages and shape immune responses. Abnormal platelet behavior during dengue infection may play a significant role in modifying lymphocyte, monocyte and granulocyte function. When platelet-leukocyte interactions were quantified *in vivo*, macrophages/monocytes appeared to be the most commonly associated cell lineage with platelets (Onlamoon et al., 2010), with a majority of these monocyte-platelet aggregates expressing activation marker CD62P (Onlamoon et al., 2010). This data is reminiscent of other reports linking activated monocytes to disease pathology in humans (Mustafa et al., 2001; Bozza et al., 2008; Durbin et al., 2008).

**Figure 3:**
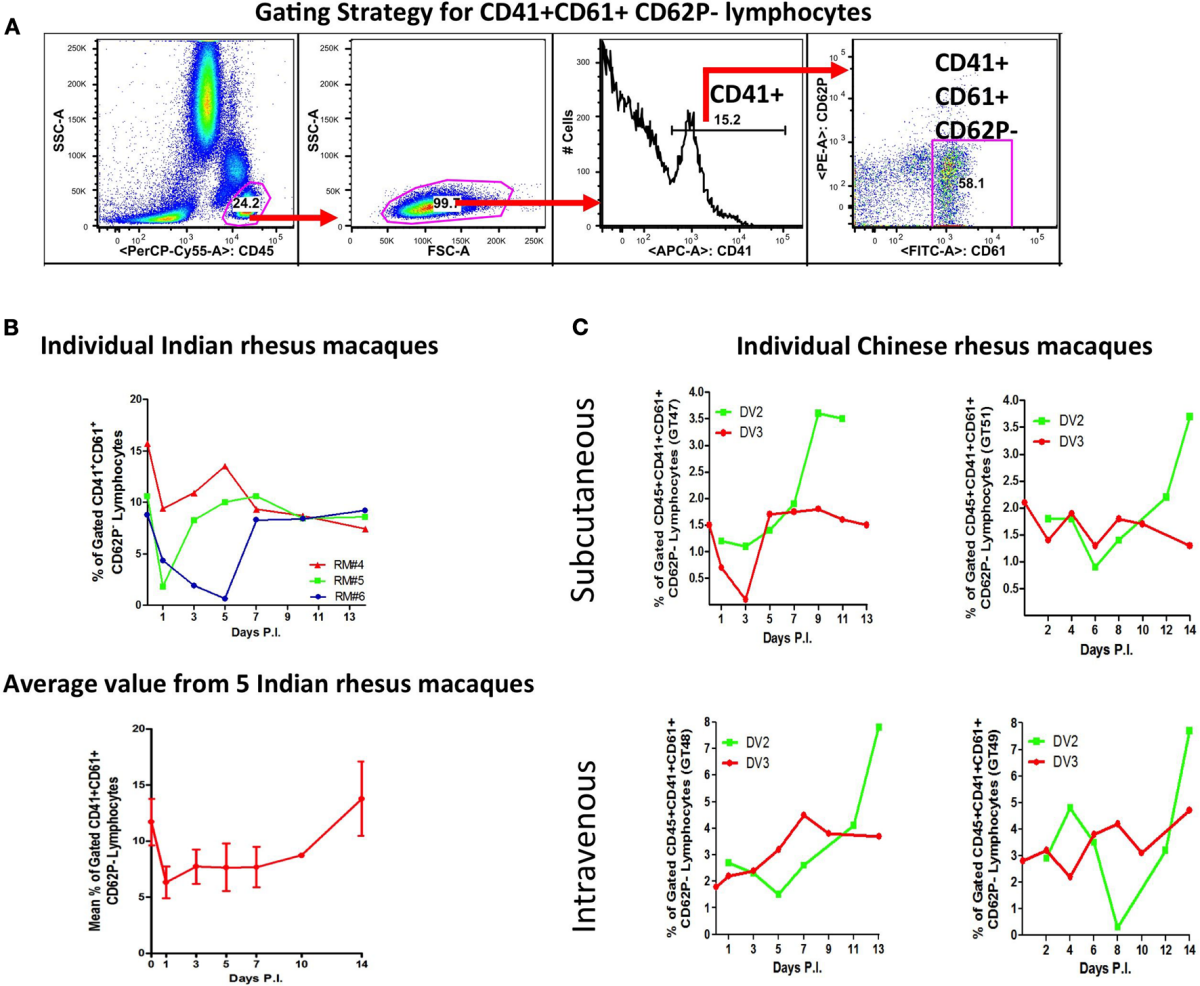
**Dynamics of lymphocyte-platelet aggregates (LymPA) during DV infection.** Indian and Chinese rhesus macaques were infected as detailed in Figure 1. In addition, the Chinese macaques were challenged 2 months later with DV3 strain Hawaii. Peripheral blood samples obtained on Days 1 through 14 were subjected to flow cytometric analysis with CD45, CD41, CD61, and CD62P fluorescent antibodies. The frequencies of CD45^+^CD41^+^CD61^+^CD62P^−^ cells over time is graphed. **(A)** Panels to illustrate the gating strategy employed to analyze lymphocyte-platelet aggregates (LymPA). **(B)** The kinetics of LymPA in Indian rhesus macaques. The top graph displays LymPA frequencies from 3 individual macaques and the bottom graph, the average population frequency from 5 primates. The LymPA population is down-regulated during DV infection in Indian rhesus macaques. **(C)** LymPA kinetics in subcutaneously and intravenously infected Chinese rhesus macaques during primary DV2 (green line) and secondary DV3 infection (red line). The frequency of LymPA increases late after primary but not after secondary infection.

Platelets binding to neutrophils and lymphocytes were less frequent (Figures 3A–C) (Onlamoon et al., 2010). Only about 20–40% of neutrophils were bound with platelets, with 30–60% expressing CD62P. However, the extend of neutrophil-platelet aggregatation may be underestimated, since these cells are short-lived and other markers for neutrophil (CD11b and CD66b) and platelet (CD154, cleaved PAR1, CD63) activation were not tested (Heijnen et al., 1999; Claytor et al., 2003; Kinhult et al., 2003; Sprague et al., 2008). Lymphocyte-platelet aggregation occurred the least (Figures 3B,C). This was examined with Indian and Chinese rhesus macaques during primary DV2 (16681) infection and in Chinese macaques during secondary DV3 (Hawaii) infection (Figures 3B,C respectively). Since the dominant phenotype of the lymphocyte-platelet aggregate (LymPA) population was CD62P negative, this was the only population evaluated. Chinese and Indian macaques have different baseline levels of CD41+CD61+CD62P− lymphocytes, approximately 2% and 12%, respectively (Figures 3B,C). The average response from 5 Indian macaques suggests that the LymPA population is down-regulated (to about 7%) during infection but returns to normal levels after viral clearance (Figure 3B). In Chinese macaques, there appeared to be higher LymPA frequencies with the IV-inoculated monkeys, ranging up to 8% but only as high as 4% in SC-inoculated primates (Figure 3C). There was a late phase expansion of this population after primary but not after secondary infection. The functional significance of such changes is unclear at the present, but it would be interesting to compare these findings with other viral infections, like influenza, which produce robust long-lived B cell memory responses (Ikonen et al., 2010; Li et al., 2012). It remains to be seen whether this observation represents a common immune phenomenon or a DV specific response, which would potentially open a new line of investigation.

## Potential refinements to the coagulopathy monkey model

### Virus selection

While the data obtained with our rhesus macaque model appears promising, many parameters remain to be examined and refined. Arguably, the most important factor to evaluate is different strains. The viruses we used had been propagated extensively in cell culture, and thus the next step will be to evaluate primary DV strains, which are considered more capable of inducing pathology. Interestingly, the earliest DV studies (pre-1940s) in primates were conducted with human-derived virus that had never been propagated through cell culture (Lavinder and Francis, 1914; Chandler and Rice, 1923; Blanc et al., 1929; Simmons et al., 1931), yet these investigations induced minimal overt disease. The human-derived Hawaiian and New Guinea strains from Sabin's work were pathogenic in humans (when inoculated intradermally) but demonstrated no pathology in Rosen's study when inoculated into various primate species via a subcutaneous or intraperitoneal route (Sabin, 1952; Rosen, 1958). In recent studies, a large number of the strains employed were recent clinical isolates minimally passaged *in vitro* (Freire et al., 2007; Omatsu et al., 2011; Pamungkas et al., 2011; Yoshida et al., 2012). While these viruses are often close in sequence to the original isolate, these strains are not necessarily the most virulent or capable of achieving the targeted pathology in primates (Omatsu et al., 2011) and may require further evaluation before use *in vivo*.

The major drawbacks of primate models are the logistics and cost. Ideally one would perform preliminary experiments and evaluate strain virulence through a screening tool before *in vivo* studies with NHPs. Virulence could be assessed by testing the induction of disease in the humanized mouse or potentially by growth characteristics in monkey whole BM. Alternatively, passage of dengue in organisms (humanized mice or rhesus macaques) may ensure that the strain is more fit for these studies. It has been suggested that mouse-passaged viruses are more capable at causing viremia in NHPs than *in vitro*-passaged strains (Scherer et al., 1972).

Considering the viruses that have already been tested in NHPs, a select few appear promising for future studies. WP-74 (DV1) and S16803 (DV2) caused extreme lethargy in owl monkeys (Schiavetta et al., 2003) but not in cynomolgus (Koraka et al., 2007) or rhesus macaques (Ajariyakhajorn et al., 2005; Robert Putnak et al., 2005). Besides the 16681 DV2 virus, strains 49313 (DV1), 16007 (DV1), and 43283 (DV4) were associated with hemorrhage in previous studies (Halstead et al., 1973b; Scherer et al., 1978). Testing these strains in our Indian macaque model could lead to a more frequent presentation of coagulopathy and models for 3 of the 4 dengue serotypes. For future preclinical vaccine and drug studies, one strain of each serotype that can induce easily quantifiable disease will be needed for better vaccine evaluation.

### Other parameters

A number of additional parameters may be manipulated in rhesus macaques that could amplify disease severity. Factors from infected mosquito saliva may potentiate the virus in down-modulating immune responses during the initiation of infection and help raise peak titer levels (Cox et al., 2012; Reagan et al., 2012; Surasombatpattana et al., 2012; Le Coupanec et al., 2013). Mosquito inoculation of DV into NHPs was modeled long ago without inducing much disease (Simmons et al., 1931). However, a number of confounding factors (preexisting immunity, inoculum quality, etc.) were not accounted for in these studies, indicating that this approach is worth revisiting.

Modulation of *in vivo* cell populations with drug treatments has rarely been attempted (Marchette et al., 1980; Yoshida et al., 2012). Potential treatment of macaques with megakaryocytic growth factors, like thrombopoetin, could increase the number of early permissive targets and enhance peak viral load if indeed megakaryocytes are the primary replication site for DV (Nakorn et al., 2003). General immunosuppression has been attempted but led to chronic viremia, which does not mimic human DV disease (Marchette et al., 1980). Depletion of macrophages, neutrophils or other innate immune responders may enhance titers by altering the dynamics of viral clearance. One previous attempt at CD16+ natural killer cell depletion did not modulate virus titers (Yoshida et al., 2012), although such depletions are generally partial at best. Additionally, various inoculum sizes and alternative inoculation routes may be tested. The intradermal inoculation route was suggested to lead to better virus tissue distribution, but did not result in better dissemination to the BM (Pamungkas et al., 2011). Characterization of these parameters are necessary for the further refinement of the coagulopathy disease animal model.

## Host characteristics or genetic factors that increase susceptibility to coagulopathy

Epidemiological studies of dengue patient characteristics, including age, sex and genetic polymorphisms have been frequently studied, but none of these findings have been validated in animal models (Loke et al., 2001; Stephens et al., 2002; Cordeiro et al., 2007; Kalayanarooj et al., 2007; Soundravally and Hoti, 2007; Stephens, 2010). In humans, the age of greatest susceptibility to disease is seen in young adults (Tsai et al., 2012). In our Indian rhesus macaques, we have evaluated age as a contributing factor to viremia by comparing the titers of DV when propagated in whole BM *in vitro* (unpublished data). However, no difference was noted in virus growth kinetics or magnitude related to age of BM donors (*n* = 11), which spanned 2–15 years of age. *In vivo*, anecdotal observations suggested that coagulopathy appeared to be more extensive in older female macaques when compared to young males, which were the populations included in the study, although sample size was too low to be conclusive. This nevertheless raises an interesting question about the potential for host factors contributing to the severity of symptoms.

Various MHC alleles, blood group and platelet antigens have been found to be associated with dengue disease and protection (Kalayanarooj et al., 2007; Soundravally and Hoti, 2007; Alagarasu et al., 2013; Weiskopf et al., 2013). Although in general these associations are weak as biomarkers of disease. One of our goals is to assess gene alleles involved with regulating platelet activation and the coagulatory cascade e.g., *HPA1*, *HPA2* for association with disease presentation. Available techniques, such as *Macaca mulatta* typing and gene expression analyses, will need to be an integral part of future experiments with the rhesus monkey model to facilitate identification of genetic factors involved with dengue-induced abnormal coagulation.

## Conclusion

The induction of disease symptoms upon the inoculation of DV in primates has been an elusive objective. Recently a coagulopathy disease model was developed using the serotype 2 strain 16681 injected intravenously into Indian rhesus macaques. We submit that this approach provides a strategy for detailed investigation of the mechanisms potentially involved in DHF. Moreover, the model provides an attractive algorithm for testing the efficacy of preventative vaccines and therapeutics that not only limit virus replication but also prevent disease development *in vivo*. Various host and viral parameters can begin to be evaluated *in vivo* to help us gain a better understanding of dengue biology and disease pathogenesis. Can pathology be induced in other NHPs by switching to the intravenous route? Will different virus strains promote coagulopathy, or other symptoms? Can we alter other parameters and achieve a more severe disease model? The establishment of this new rhesus macaque infection model has proved insightful on ways to improve disease presentation in primates.

## Human subject and animal research

Use of deidentified human BM was provided by Emory Hospital and approved by the Emory University Internal Review Board. Investigations with rhesus macaques were approved by Yerkes and Tulane IACUCs and conducted at either the Yerkes or Tulane National Primate Research Centers. Research was performed in accordance with institutional and national guidelines and regulations.

### Conflict of interest statement

The authors declare that the research was conducted in the absence of any commercial or financial relationships that could be construed as a potential conflict of interest.
